# Cytokine release syndrome after allogeneic hematopoietic stem cell transplantation using posttransplant cyclophosphamide: current understanding and management

**DOI:** 10.3389/fimmu.2026.1642583

**Published:** 2026-05-04

**Authors:** Jiasheng Wang, Kirti Arora, Marcos de Lima

**Affiliations:** 1 Division of Hematology, Department of Medicine, The Ohio State University Comprehensive Cancer Center, Columbus, OH, United States; 2 Department of Internal Medicine, Cleveland Clinic Akron General, Akron, OH, United States

**Keywords:** cytokine release syndrome (CRS), hematopoeietic stem cell transplantation, neurotoxicicity, post transplant cyclophosphamide, tocilizumab

## Abstract

Post-transplant cyclophosphamide (PTCy) is increasingly used for graft-versus-host disease (GVHD) prophylaxis in allogeneic hematopoietic stem cell transplantation (alloHCT), across both haploidentical and HLA-matched donor settings. However, PTCy-based alloHCT is associated with post-transplant cytokine release syndrome (CRS), an early inflammatory toxicity driven by donor T-cell alloreactivity. Mechanistically distinct from chimeric antigen receptor (CAR) T-cell therapy-associated CRS, post-transplant CRS is driven by conditioning-induced tissue injury and donor T-cell alloreactivity. The incidence of CRS varies by donor type, occurring in approximately 10% of matched sibling, 20 to 75% of matched unrelated, and 80 to 90% of haploidentical donor transplants. Risk factors include peripheral blood grafts, HLA class II mismatch, and high-intensity conditioning. Severe CRS has been linked to delayed engraftment, increased acute GVHD, decreased chronic GVHD, and may contribute to non-relapse mortality through mechanisms such as third-spacing, infectious complications, and neurologic toxicity. Tocilizumab is effective for CRS treatment, though its impact on subsequent GVHD risk requires further study. Early initiation of calcineurin inhibitors and use of pre-transplant anti-thymocyte globulin have shown promise in reducing CRS incidence. CRS-associated neurotoxicity, resembling immune effector cell-associated neurotoxicity syndrome (ICANS), is increasingly recognized, particularly among patients with severe CRS, and can result in delayed, fatal encephalopathy. In summary, post-transplant CRS is a clinically significant complication of PTCy-based alloHCT. Optimizing prophylaxis and management strategies is essential to mitigate its impact on transplant outcomes.

## Introduction

Post-transplant cyclophosphamide (PTCy) is increasingly used in allogeneic hematopoietic stem cell transplantation (alloHCT) as an effective prophylaxis for graft-versus-host disease (GVHD). Typically administered on days +3 and +4 post-transplant, high-dose PTCy selectively eliminates alloreactive T cells while sparing regulatory T cells (Tregs), thereby mitigating both acute and chronic GVHD (1). Originally developed for haploidentical transplants (2), PTCy has since been successfully applied to mismatched donor grafts (3). Recent randomized trials demonstrate that PTCy is also effective in HLA-matched unrelated and related donor transplants, achieving comparable survival outcomes with lower rates of chronic GVHD compared to calcineurin inhibitor-based GVHD prophylaxis (4). Its ease of use and ability to expand donor availability have contributed to its growing adoption in transplant protocols worldwide.

However, transplantation approaches incorporating delayed post-infusion immunosuppression, such as those using PTCy, are susceptible to early cytokine release syndrome (CRS). CRS is a systemic inflammatory condition driven by immune activation and cytokine release, clinically characterized by non-infectious fever and, in more severe cases, hypoxia, hemodynamic instability and organ dysfunction. This was first reported in the initial clinical trial by the Johns Hopkins group in 2002, where it occurred in 5 of 13 patients between posttransplant days 1 and 3, and was initially classified as neutropenic fever without further etiologic investigation (5). Broader recognition of this phenomenon emerged around 2015, when peripheral blood stem cells (PBSCs) replaced bone marrow as the graft source, leading to a higher incidence and greater severity of early post-transplant fever (6–8). Initially considered unique to haploidentical transplantation and termed “haplo-storm” or “haplo-fever,” it has since been recognized that post-transplant CRS also occurs in transplants from matched donors (9). In addition, neurotoxicity — another inflammation-related toxicity observed in cellular therapies such as CAR T-cell therapy — has been identified in patients with severe CRS following PTCy (10).

In this review, we will examine current evidence regarding the mechanism, incidence, risk factors, clinical impact, and strategies for prophylaxis and management of post-transplant CRS.

## Mechanisms of post-transplant CRS

A hallmark of PTCy-based HCT is the delayed administration of high-dose cyclophosphamide on days +3 and +4, a strategy derived from animal studies demonstrating that this timing optimally induces immune tolerance (11). In addition, the initiation of immunosuppressants calcineurin inhibitors and mycophenolate is intentionally delayed until after cyclophosphamide, as preclinical data indicate that calcineurin inhibitors can interfere with cyclophosphamide-induced tolerance (12). As a result, infused alloreactive T cells remain unmodulated during the first two days post-transplant, a period that coincides with the occurrence of CRS. While the mechanisms of CRS are well characterized in the context of CAR T-cell therapy (13), they remain less well understood in the post-transplant setting, and emerging evidence suggests important mechanistic differences.

In CAR T-cell therapy-associated CRS, the initial cytokine surge is triggered by lymphodepletion chemotherapy followed by activation of CAR T cells upon antigen recognition via the CAR. This leads to the release of interferon-gamma (IFN-γ) and tumor necrosis factor-alpha (TNF-α), which in turn stimulate bystander myeloid cells, such as monocytes and macrophages, to secrete additional proinflammatory cytokines, including interleukin-1 (IL-1) and interleukin-6 (IL-6) (14, 15). This amplifies CAR T-cell activation, creating a self-perpetuating cycle of cytokine release and systemic inflammation.

However, CRS in the post-transplant setting likely has different mechanisms. First, T-cell activation occurs via the conventional T-cell receptor (TCR) through alloantigen recognition, rather than the more potent CAR-mediated activation. The occurrence of post-transplant CRS, though less frequent, even in HLA-matched transplants suggests that TCR activation through minor H antigens can also trigger CRS. Second, post-transplant CRS is characterized by elevations of T-cell-derived cytokines. Serial cytokine profiling during CRS shows early, transient increases in multiple cytokines beginning on day +1, peaking between days +2 and +4, and normalizing after PTCy administration. Across studies, IFN-γ, IL-6, and soluble IL-2 receptor show the most consistent elevations, with variable increases in IL-2, IL-8, IL-10, IL-17, and TNF across different studies (16, 17). In contrast to CAR T-cell CRS, monocyte-related cytokines such as IL-1β and monocyte chemotactic protein-1 (MCP-1) are not elevated, indicating limited monocyte involvement (18). The lack of bystander monocyte activation may explain why post-transplant CRS, unlike CAR T-cell CRS, lacks a self-perpetuating cytokine cycle, and is therefore rarely refractory, typically resolving after PTCy administration. Third, CRS is more frequent and severe with higher-intensity conditioning regimens (19), suggesting that tissue damage and the associated release of damage-associated molecular patterns (DAMPs) play a key role in T-cell activation. Moreover, intensive conditioning may reduce the cytokine sink, increasing the availability of homeostatic cytokines and thereby potentiating CRS, analogous to the role of lymphodepletion in CAR T-cell therapy (20). Fourth, class II HLA mismatch has consistently been shown to be a strong predictor of CRS, whereas class I mismatch is not (19, 21), implicating CD4+ T cells as central drivers of CRS. Supporting this hypothesis, immune reconstitution analyses demonstrate higher frequencies of both CD4^+^ Tcon and CD4^+^ Treg populations at the first post-transplant sampling time point (approximately day +30 in the study) in patients who developed CRS, with no persistence at later time points (22).

**Figure 1:**
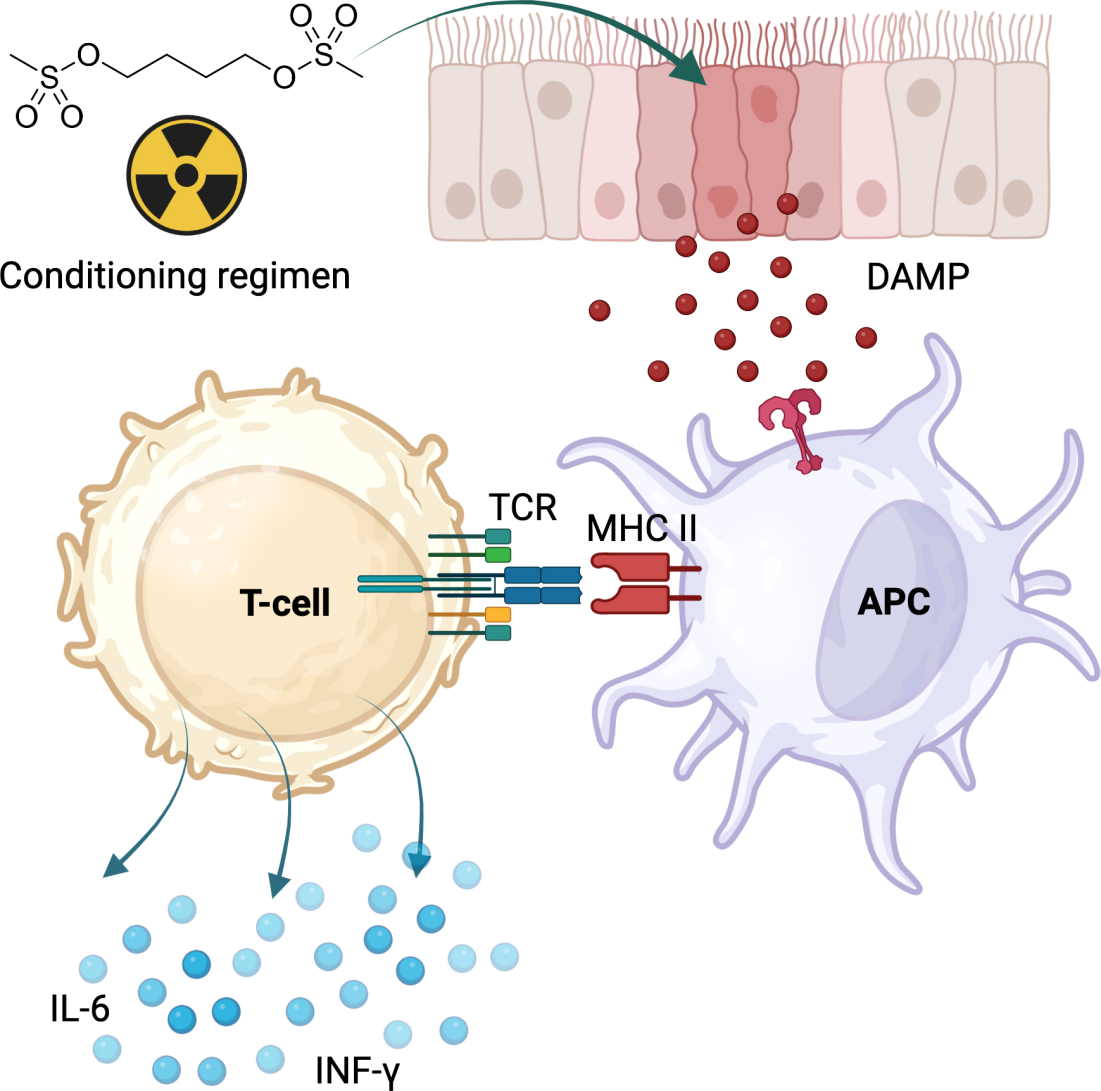
Proposed mechanism of CRS following alloHCT with PTCy. Conditioning chemotherapy and radiation induce tissue damage, triggering the release of damage-associated molecular patterns (DAMPs). These DAMPs activate antigen-presenting cells (APCs) and other innate immune cells, which in turn stimulate alloreactive T cells, primarily through interactions between the T-cell receptor (TCR) and MHC molecules, particularly MHC class II. Activated T cells then secrete proinflammatory cytokines, such as IL-6 and IFN-γ, driving the development of CRS.

Collectively, these findings suggest that post-transplant CRS is driven by conditioning-induced tissue injury and donor T-cell alloreactivity, resulting in T-cell-mediated cytokine release and expansion (**Figure 1**).

## Incidences and risk factors of CRS

The incidence of CRS varies according to the degree of HLA matching. (**Table 1**) Two common grading systems are used to grade CRS: the Lee criteria (23) and the ASTCT criteria (24). These two systems are generally comparable, though the latter is a simplified version of the former.

**Table 1:** Incidences of CRS based on donor types.

Studies	Total patients	Stem cell source	CRS grading criteria	CRS incidence
Matched Sibling Donor				
McCurdy et al., 2018 (19)	183	BM	No grading	• Overall: 13%
Solan et al., 2020 (9)	39	Majority PB	Lee	• Overall: 10% • Grade 3-5: 0%
von dem Borne et al., 2024 (25)	19	PB	ASTCT	• Overall: 21% • Grade 3-5: 0%
Benavente et al., 2025 (26)	196	PB or BM	ASTCT	• Overall: 8% • Grade 3-5: 0%
Matched Unrelated Donor				
McCurdy et al., 2018 (19)	115	BM	No grading	• Overall: 24%
von dem Borne et al., 2024 (25)	68	PB	ASTCT	12/12 match • Overall: 38% • Grade 3-5: 0% 10/10 match • Overall: 75% • Grade 3-5: 2%
Benavente et al., 2025 (26)	187	PB or BM	ASTCT	• Overall: 23% • Grade 3-5: 0%
Mismatched Unrelated Donor				
von dem Borne et al., 2024 (25)	38	PB	ASTCT	• Overall: 87% • Grade 3-5: 26%
Benavente et al., 2025 (26)	22	PB or BM	ASTCT	• Overall: 32% • Grade 3-5: 0%
Haploidentical Donor				
Abboud et al., 2016 (17)	75	PB	Lee	• Overall: 87% • Grade 3-5: 12%
Arango et al., 2017 (27)	40	PB	Lee	• Overall: 83% • Grade 3-5: 0%
McCurdy et al., 2018 (19)	374	BM	No grading	• Overall: 53%
Raj et al., 2018 (28)	66	PB or BM	Lee	• Overall: 73% • Grade 3-5: 11%
Imus et al., 2019 (29)	146	PB	Lee	• Overall: 89% • Grade 3-5: 17%
Mariotti et al., 2019 (21)	102	PB or BM	Lee	• Overall: 86% • Grade 3-5: 15%
Solh et al., 2019 (30)	172	PB or BM	Lee	• Overall: 81% • Grade 3-5: 1%
Solan et al., 2020 (9)	107	Majority PB	Lee	• Overall: 76% • Grade 3-5: 1%
Abboud et al., 2021 (31)	451	PB or BM	Lee	• Overall: 90% • Grade 3-5: 17%
Modi et al., 2021 (32)	98	PB	ASTCT	• Overall: 93% • Grade 3-5: 10%
Otoukesh et al., 2022 (33)	271	PB	ASTCT	• Overall: 93% • Grade 3-5: 5%
Shapiro et al., 2023 (22)	169	PB or BM	ASTCT	• Overall: 58% • Grade 3-5: 1%
Benavente et al., 2025 (26)	147	PB or BM	ASTCT	• Overall: 80% • Grade 3-5: 1%

CRS, cytokine release syndrome. ASTCT, American Society for Transplantation and Cellular Therapy. PB, peripheral blood. BM, Bone Marrow. MAC, Myeloablative Conditioning. MRD, Matched Related Donor. MUD, Matched Unrelated Donor. NMA, Non Myeloablative. Haplo, Haploidentical.

1. In matched sibling donor alloHCT, the incidence of CRS is approximately 10% (9, 19, 25, 26), with no cases of severe CRS (Grade 3 to 5) reported in these cohorts.
2. In 8/8 HLA-matched unrelated donor (MUD) alloHCT, the reported incidence of CRS ranges widely, from 20% to 75% (19, 25, 26), likely due to the presence of HLA-DPB1 mismatches in some patients — a known risk factor for CRS. Notably, severe CRS remains rare in this group, with an incidence of 0–2%.
3. In mismatched unrelated donor (MMUD) alloHCT, only two studies have reported the incidence of CRS with overall rates of 32% and 87%, respectively. This variability is likely attributable to differences in the degree of mismatch and the use of bone marrow grafts in one study, as bone marrow grafts are associated with a lower incidence of CRS (25, 26).
4. In haploidentical donor alloHCT, the overall incidence of CRS is approximately 80–90% (9, 17, 19, 21, 22, 26–33)., while the incidence of severe CRS ranges from 0 to 20%, with most studies reporting rates below 10%.

Several clinical risk factors for CRS have been consistently identified across multiple studies. (**Table 2**) These include the use of peripheral blood grafts instead of bone marrow grafts, the overall degree of HLA mismatch, and mismatches at HLA class II loci, particularly HLA-DRB1 and HLA-DPB1. Additional risk factors have been identified in some, but not all, studies. These include a high T-cell dose in the graft, active disease at the time of transplant, the use of myeloablative conditioning, and a higher pretransplant comorbidity index. For example, several studies have identified CD3^+^ cell dose as a continuous risk factor for CRS (19, 30, 33), with doses exceeding 4 × 10^8^ cells/kg significantly associated with an increased incidence of CRS (33).

**Table 2:** Risk factors for CRS.

Studies	Risk factors
McCurdy et al., 2018 (19)	Haploidentical • Mismatch in DRB1 or DPB1 • Higher T-cell count HLA-matched: • Higher T-cell count • Pretransplant active disease or minimal residual disease • Higer HCT-CI score
Raj et al., 2018 (28)	• PB grafts
Imus et al., 2019 (29)	• Mismatch in DRB1 • Older recipient age • Previous radiation therapy
Mariotti et al., 2019 (21)	• PB graft • Mismatch in DRB1 • Pretransplant active disease
Solh et al., 2019 (30)	• Overall degree of HLA mismatch • PB graft • Myeloablative conditioning • Higher T-cell count • Higher stem cell count
Solan et al., 2020 (9)	• PB graft • In haploidentical transplant, higher median total nucleated cell count
Abboud et al., 2021 (31)	• PB graft • Recipient CMV seropositivity • Prior transplant • Higher HCT-CI score • Donor–recipient sex mismatch
Modi et al., 2021 (32)	• No risk factor identified
Otoukesh et al., 2022 (33)	• Overall degree of HLA mismatch • Higher T-cell dose • Older recipient age • Reduced intensity conditioning
Shapiro et al.,2023 (22)	• PB graft • Higher stem cell dose
von dem Borne et al., 2024 (25)	• Overall degree of HLA mismatch • Non-permissive DPB1-mismatch in MUD transplant • DRB1-mismatch in 9/10 matched group
Benavente et al., 2025 (26)	• Overall degree of HLA mismatch • In haploidentical transplant, peripheral blood rather than bone marrow donor source

NMA, Non Myeloablative. Haplo, Haploidentical. BMT, Bone Marrow Transplant. GVHD, graft-versus-host disease. MAC, Myeloablative Conditioning. RIC, Reduced Intensity Conditioning.

## Impact of CRS on outcomes

The occurrence of CRS has been shown to adversely affect short-term outcomes, including engraftment. Severe CRS has been associated with delayed neutrophil and platelet recovery, with neutrophil engraftment delayed from a median of approximately 16 days to 21–29 days, and platelet engraftment from around 25 days to beyond 30 days (17, 29, 31). Interestingly, Shapiro et al. demonstrated that in patients with CRS, the number of T-cells, including CD4+ Tcon, CD4+ Treg, and CD8+ T cells temporarily increased one month after alloHCT compared to patients without CRS, followed by a return to baseline levels (22). This transient T-cell reconstitution, likely driven by cytokine stimulation, may contribute to the impact of CRS on long-term outcomes.

In haploidentical transplantation, the association between CRS and GVHD appears to be severity dependent. In the study reported by Raj et al., severe CRS showed a severity-dependent effect: while CRS grade ≥2 was associated only with a trend toward increased grade III–IV acute GVHD, CRS grade ≥3 was significantly associated with a markedly higher incidence of grade III–IV acute GVHD (28). In contrast, in studies in which CRS was uniformly low grade, including those reported by Solán et al. and McCurdy et al., CRS was associated with an increased incidence of grade II acute GVHD, but not with high-grade (III–IV) acute GVHD (9, 19). Notably, several studies have reported that CRS in the haploidentical setting is associated with a reduced risk of moderate-to-severe chronic GVHD (19, 26, 29, 31, 32). The mechanisms underlying this paradoxical finding remain unclear. It has been speculated that early immune activation and effector T-cell expansion may increase the risk of acute GVHD, while the concurrent expansion of Tregs and cytokine-driven exhaustion of alloreactive T cells could reduce the risk of chronic GVHD — a mechanism similar to the known protective effects of PTCy in reducing chronic GVHD. In contrast, in the HLA-matched donor setting, CRS is typically mild and has not been associated with an increased risk of either acute or chronic GVHD (26).

Regarding non-relapse mortality (NRM), several studies have shown that severe, but not mild, CRS is associated with an increased risk of NRM (17, 21, 29, 31–33). Importantly, affected patients typically do not succumb to CRS itself, but rather to complications such as infections, encephalopathy, or multi-organ failure (29, 32). Capillary leak and fluid retention, which are common during severe CRS, can lead to significant third-spacing of fluids, tissue edema, and impaired organ perfusion, ultimately contributing to multi-organ dysfunction and failure. Only a few studies have examined the association between CRS and relapse risk, and all have consistently reported a decreased risk of relapse in patients who experienced CRS (22, 26, 31). This is a somewhat surprising finding, especially given that CRS is also associated with a reduced risk of chronic GVHD. Larger studies are needed to confirm this observation and to further elucidate the underlying mechanisms. For overall survival (OS), approximately half of the studies have reported worse OS associated with severe CRS, while the others did not observe a significant difference (**Table 3**).

**Table 3:** Impact of CRS on outcomes.

Studies	Engraftment	GVHD	NRM	Relapse	OS
Abboud et al., 2016 (17)	• Delayed neutrophil engraftment in severe CRS • Not associated with platelet engraftment	No difference	Higher in Grade 3–4 CRS	N/A	Worse in Gr3–4 CRS
Arango et al., 2017 (27)	N/A	N/A	N/A	No difference	No difference
McCurdy et al., 2018 (19)	No difference	HLA-matched: • Higher risk of Gr III-IV aGVHD • Lower risk of cGVHD Haploidentical: • Higher risk of Gr II aGVHD	No difference	No difference	No difference
Raj et al., 2018 (28)	N/A	Higher Gr III-IV aGVHD in severe CRS	No difference	No difference	No difference
Imus et al., 2019 (29)	• Delayed neutrophil engraftment in severe CRS • Delayed platelet engraftment in severe CRS	Lower risk of moderate-to-severe cGVHD in severe CRS	Higher in severe CRS	N/A	Worse in severe CRS
Mariotti et al., 2019 (21)	No difference	No difference	Higher in severe CRS	N/A	Worse in severe CRS
Solh et al., 2019 (30)	No difference	No difference	No difference	No difference	No difference
Solan et al., 2020 (9)	No difference	Haploidentical: • Higher risk Gr II-IV aGVHD HLA matched: • No difference	No difference	No difference	No difference
Abboud et al., 2021 (31)	• Delayed neutrophil engraftment in severe CRS • Delayed platelet engraftment in severe CRS	Lower risk of cGVHD in severe CRS	Higher in severe CRS	Decreased in mild or severe CRS	Worse in severe CRS
Modi et al., 2021 (32)	No difference	Lower risk of cGVHD in severe CRS	Higher in severe CRS	No difference	Worse in severe CRS
Otoukesh et al., 2022 (33)	No difference	No difference	Higher in severe CRS	No difference	Worse in severe CRS
Shapiro et al., 2023 (22)	Enhanced T-cell engraftment	Increased risk of cGVHD	No difference	Decreased	No difference
von dem Borne et al., 2024 (25)	N/A	No difference	Increased in Grade 2–3 CRS	No difference	Worsened in Grade 2–3 CRS
Benavente et al., 2025 (26)	No difference	Haploidentical: • Lower risk of severe cGVHD HLA matched: • No difference	Haploidentical • Lower HLA matched: • No difference	Haploidentical • Decreased HLA matched: • No difference	Haploidentical • Improved OS HLA matched: • No difference

CRS, cytokine release syndrome. GVHD, graft-versus-host disease. NRM, non-relapse mortality. DFS, disease-free survival. OS, overall survival. Gr, grade. MAC, Myeloablative Conditioning. MRD, Matched Related Donor. MUD, Matched Unrelated Donor. NMA, Non Myeloablative.

## Management of CRS

### Treatment

Given the marked elevation of IL-6 in post-transplant CRS and the experience gained from managing CAR T-related CRS, tocilizumab has been used to mitigate symptoms prior to PTCy administration. It has consistently demonstrated efficacy in resolving CRS. Abboud et al. first reported its use in 7 patients, including 3 with severe CRS and 4 with significant comorbidities; in all cases, CRS resolved within 48 hours (17). Similarly, Yao et al. reported that 11 of 115 patients with CRS received tocilizumab on either Day 3 or Day 4, with complete resolution of symptoms in all treated patients (34). Despite the effectiveness, the use of tocilizumab varies across institutions. Some centers initiate treatment for low-grade CRS in patients with significant comorbidities, while others reserve it for severe cases, given the high rate of CRS resolution following PTCy alone (31).

However, potential side effects of tocilizumab use should be mentioned. One concern is the potential increased risk of infection. CRS itself is associated with higher rates of viral, bacterial, and invasive fungal infections (35, 36). While there is a theoretical concern that tocilizumab could further elevate this risk (37), such an effect has not been observed in its use for CAR T-related CRS (38). Indeed, a study by Togni et al. comparing 49 patients received tocilizumab and 186 patients who did not, after adjusting for the CRS grade, the two groups had similar rates of blood stream infection (39). Another concern arises from the role of IL-6 in T-cell activation and expansion. Blunting IL-6 signaling with tocilizumab may affect the immunomodulatory efficacy of subsequent cyclophosphamide and compromise GVHD prevention. On the other hand, tocilizumab has demonstrated efficacy in the treatment of acute GVHD (40), and, in a phase III trial, showed a nonsignificant trend toward lower incidence of grade II-IV aGVHD In the HLA-matched unrelated donor transplant setting (41). However, in a small study by Yao et al., patients who received tocilizumab for CRS had nearly twice the incidence of chronic GVHD compared to those who did not (34). Larger, adequately powered studies are needed to determine whether this association is reproducible and clinically meaningful.

Corticosteroids are commonly used in the management of CAR T-related CRS, including in prophylactic settings, as studies have shown that steroids do not significantly impair CAR T-cell function (42, 43). However, their use in the early post-transplant setting requires caution due to concerns about potential effects on stem cell engraftment and reduction of graft-versus-leukemia activity. In the context of PTCy, the impact of steroids on alloreactive T-cell proliferation, and the potential to interfere with the efficacy of PTCy in eliminating donor-reactive T cells, is not well understood. As a result, steroids are generally avoided prior to PTCy administration (44, 45). Nevertheless, steroids have been reported to successfully treat severe post-transplant CRS that persists beyond cyclophosphamide (31, 45).

### Prophylaxis

Given that post-transplant CRS is mediated by alloreactive T-cells, several studies have explored whether earlier initiation of immunosuppressants can reduce CRS without compromising the efficacy of GVHD prevention. In a study by Bacigalupo et al., cyclosporin and mycophenolate were started on day 0 and day +1, with PTCy administered on day +3 and +5 (46). Among 425 patients receiving haploidentical bone marrow grafts, low incidences of grade III-IV aGVHD (3%) and moderate-severe cGVHD (18%) were reported; however, CRS incidence was not documented (46). In another study by Kurita et al. in haploidentical alloHCT, cyclosporin and mycophenolate were initiated on day -1, with PTCy given on day +3 and +5. CRS occurred in only 20% of patients, all of whom experienced grade 1 CRS. The incidences of grade III-IV aGVHD and moderate-severe cGVHD were 6% and 11%, respectively (47). Similarly, in a study by Tang et al., tacrolimus and mycophenolate were started on day -1 and day 0, with PTCy administered on day +3 and +4 (48). Compared to a historical control where both agents were started on day +5, earlier initiation resulted in a markedly lower incidence of all-grade CRS (32% vs 82%) and grade ≥2 CRS (16% vs 57%). Notably, early initiation was also associated with a significantly lower incidence of grade II-IV aGVHD (29% vs 50%). Finally, a retrospective study from the European Society for Blood and Marrow Transplantation (EBMT) showed that starting cyclosporin on day 0 and mycophenolate on day +1, compared to both on day +5, was associated with significantly reduced grade II-IV aGVHD (18% vs 39%) with comparable rates of extensive cGVHD (9% vs 7%) (49). Given these promising results of reduced CRS and potentially reduced acute GVHD, the strategy of early initiation of calcineurin inhibitors and mycophenolate warrants further investigation.

Another strategy to control alloreactive T-cells is to administer low-dose pre-transplant anti-thymocyte globulin (ATG) combined with PTCy, a regimen that is increasingly being studied in Europe for haploidentical alloHCT. Due to the long half-life of ATG, alloreactive T-cell proliferation is suppressed prior to PTCy administration. Retrospective studies have shown that this combination is associated with a lower incidence of chronic GVHD without increasing the risk of infection or relapse, compared to PTCy alone (50). In one study of 51 patients treated with this regimen, only 10% developed CRS, all of which were grade 1 or 2 (51). Therefore, these findings support further development of dual T-cell depletion with ATG and PTCy for GVHD prophylaxis in haploidentical or mismatched alloHCT.

### How we manage CRS

At our center, we initiate broad-spectrum antibiotics promptly at the onset of fever, even when the timing is most consistent with CRS, as patients are typically neutropenic at this stage and at high risk for infection. If the fever resolves after administration of cyclophosphamide and infectious workups remain negative, we promptly de-escalate antibiotics to prophylactic fluoroquinolone. We use tocilizumab relatively liberally, typically administering it to patients who have persistent CRS between the stem cell infusion and Day +2, Grade 2 or higher CRS, or at the first signs of CRS in patients with poor performance status, to ensure they remain in good physical condition to tolerate high-dose cyclophosphamide. However, we generally avoid administering tocilizumab if fever develops on Day +3 or +4, as we anticipate that cyclophosphamide will effectively resolve CRS.

## CRS associated neurotoxicity

Neurotoxicity, or immune effector cell-associated neurotoxicity syndrome (ICANS), is a well-recognized complication of CAR T-cell therapy. Previously termed cytokine release encephalopathy syndrome (CRES), ICANS is characterized by increased blood-brain barrier permeability driven by cytokine surges (52).

In the post-transplant setting, non-infectious neurotoxicity has also been increasingly recognized (53). However, neurotoxicity specifically related to CRS in this context has received comparatively little attention. Available data suggest that the risk of neurotoxicity increases with CRS severity. In a study by Imus et al., moderate to severe encephalopathy occurred in 13 of 25 patients (52%) with severe CRS (grade 3-5), and most of these patients required intubation due to encephalopathy (29). Similarly, Abboud et al. reported that 4 of 9 patients with grade 3–4 CRS developed altered mental status without other identifiable causes (17). In contrast, encephalopathy appears to be uncommon in lower-grade CRS; in one study, the incidence was only 1.7% among patients with grade 0–1 CRS (25). Notably, encephalopathy typically coincided with the onset of CRS but, in some cases, persisted beyond CRS resolution (17). Interestingly, von dem Borne et al. reported cases of delayed-onset neurotoxicity, resembling the delayed timing of ICANS relative to CRS observed in CAR T-cell therapy (25). Among 15 patients with CRS-related mortality, 4 deaths were attributed to encephalopathy without an identifiable cause. These cases presented with progressive confusion or somnolence at a median of 18 days post-transplant, without corresponding imaging abnormalities and without clinical improvement after discontinuation of tacrolimus — features clinically resembling ICANS (25). Given the high mortality associated with neurotoxicity, further studies are needed to better characterize its clinical manifestations and to guide optimal management strategies.

## Conclusion

The impact of posttransplant CRS on outcomes is heterogeneous and strongly dependent on transplant type and CRS severity, with limited clinical consequences in HLA-matched donor transplants but adverse effects associated with severe CRS in haploidentical and mismatched settings. Tocilizumab is effective for CRS control and is most likely to benefit patients with moderate-to-severe or persistent CRS, particularly those with limited physiological reserve, while its routine use in low-grade CRS remains uncertain. Further prospective studies are needed to define transplant-specific risks and optimize CRS-directed management strategies.
